# Preparation of an Immunoaffinity Column with Amino-Silica Gel Microparticles and Its Application in Sample Cleanup for Aflatoxin Detection in Agri-Products 

**DOI:** 10.3390/molecules18022222

**Published:** 2013-02-11

**Authors:** Fei Ma, Ran Chen, Peiwu Li, Qi Zhang, Wen Zhang, Xiaofeng Hu

**Affiliations:** 1 Oil Crops Research Institute of the Chinese Academy of Agricultural Sciences, Wuhan 430062, China; E-Mails: mafeicpu@163.com (F.M.); chen-jiaran@163.com (R.C.); zhangwen@oilcrops.cn (W.Z.); hxf5646@gmail.com (X.H.); 2 Key Laboratory of Biology and Genetic Improvement of Oil Crops, Ministry of Agriculture, Wuhan 430062, China; 3 Key Laboratory of Detection for Mycotoxins, Ministry of Agriculture, Wuhan 430062, China; 4 Laboratory of Risk Assessment for Oilseeds Products (Wuhan), Ministry of Agriculture, Wuhan 430062, China; 5 Quality Inspection and Test Center for Oilseeds Products, Ministry of Agriculture, Wuhan 430062, China

**Keywords:** aflatoxins, immunoaffinity column, amino-silica gel, microparticle conjugate, agri-products, HPLC

## Abstract

This study established an immunoaffinity column for selective extraction of aflatoxins in agri-products. Specifically, the immunoaffinity column was developed by covalently coupling monoclonal antibody 1C11 against aflatoxins to amino-silica gel microparticles and then packing these into a cartridge. The extraction conditions were thoroughly optimized in terms of loading, washing and eluting solutions. Under the optimal conditions, the immunoaffinity column had a capacity of 200 ng of aflatoxins. The detection limits (S/N = 3) for aflatoxin G_1_, B_1_, G_2_ and B_2_ were 0.03, 0.07, 0.05 and 0.09 μg·kg^−1^, and the corresponding quantification limits (S/N = 10) were 0.10, 0.25, 0.18 and 0.30 μg·kg^−1^, respectively. The recoveries of aflatoxins in samples were 90.1%–104.4% and RSDs were <4.4%. The developed method was further applied to the determination of aflatoxins in peanut, vegetable oil and tea samples, and the results indicated that peanut (26.9%), vegetable oils (28.0%) and tea (5.3%) samples were contaminated with aflatoxins, with levels ranging from 0.49 to 20.79 μg·kg^−1^.

## 1. Introduction

Aflatoxins (AFT), a group of naturally-occurring mycotoxins, are produced by many species of *Aspergillus* fungi*,* most notably *A*. *flavus* and *A*. *parasiticus*. Aflatoxin-producing members of *Aspergillus* are common and widespread in Nature. In particular, peanuts, nuts, vegetable oils, and cereals are known to often be contaminated with this class of mycotoxins [1]. Aflatoxin B_1_ (AFB_1_) has been reported to cause liver tumors in different animal species [2,3]. The European Commission has proposed to set tolerance levels at 2 μg·kg^−1^ for AFB_1_ and at 4 μg·kg^−1^ for total aflatoxins in certain foods [4]. The maximum limit (ML) set by United States and China is 20 µg·kg^−1^ in foodstuffs.

The current analytical techniques used to assay aflatoxin levels include thin-layer chromatography (TLC) [5], high performance liquid chromatography (HPLC) [6], liquid chromatography-mass spectroscopy (LC-MS) [7], LC-MS/MS [8], enzyme-linked immunosorbent assay (ELISA) [9], ion mobility spectrometry and so on [10]. Immunoaffinity utilizes the specific and reversible interaction between an antibody and antigen, providing the most powerful separation and purification of target analytes from complex matrices [11]. Various applications of immunoaffinity chromatography for toxins, veterinary drugs and pesticide residues have been reported [12,13].

In this work, we will describe a procedure for the preparation of a monoclonal antibody (MAb) using 1C11-based immunoaffinity chromatography followed by HPLC analysis of AFT in agri-product samples. The aims of this study were to: (1) prepare and identify an immunoaffinity column (IAC) employing MAb 1C11 and amino-silica gel microparticles; (2) develop optimal extraction conditions for the binding and release of antibody-bound AF Tfrom the IAC; and (3) evaluate the prepared IAC for the effective extraction of AFT from real samples.

## 2. Results and Discussion

### 2.1. Characterization of the Antibody-Amino Silica Gel Microparticles

**Scheme 1:**
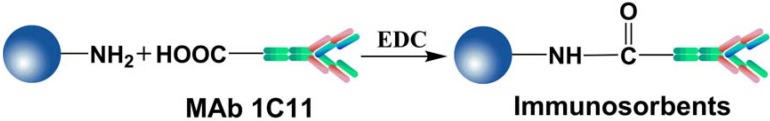
Model reaction between amino-silica gel microparticles and antibody proteins.

**Figure 1:**
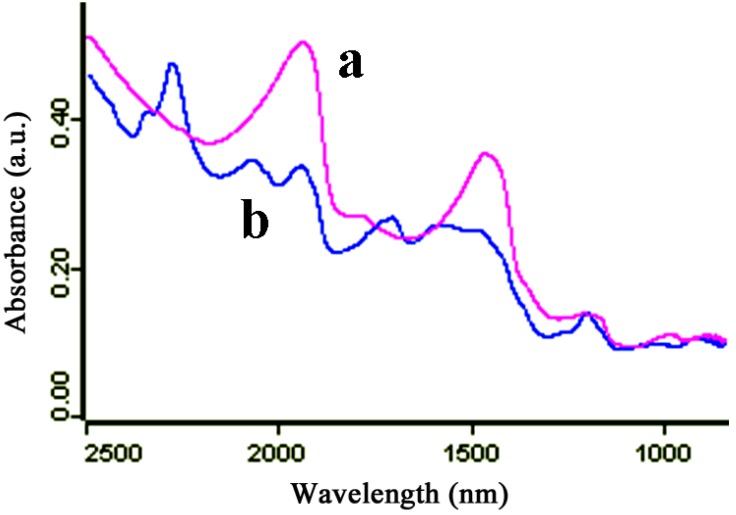
NIR spectra of amino-silica gel microparticles coupled with antibody (**a**) and amino-silica gel microparticles (**b**).

The general scheme of the reaction of amino-silica gel microparticles and MAb 1C11 is shown in Scheme 1. The immunosorbents were prepared by conjugating amino-silica gel microparticles and the carbonyl residues of MAb 1C11 using the EDC·HCl method. Figure 1 shows NIR spectra of amino silica gel microparticles, and the antibody-amino silica gel microparticle conjugate. A peak at 2280 nm in the blue curve was the result of the surface resonance of the amino silica gel microparticles. After adding MAb 1C11, the peak shifted to 1970 nm (red curve). Due to the scheme mentioned above, the peak of the carbonyl appeared at 1490 nm. According to the equation in Section 3.3, the yield of antibody-amino silica gel microparticle conjugate was 87%.

### 2.2. Loading Conditions

**Figure 2:**
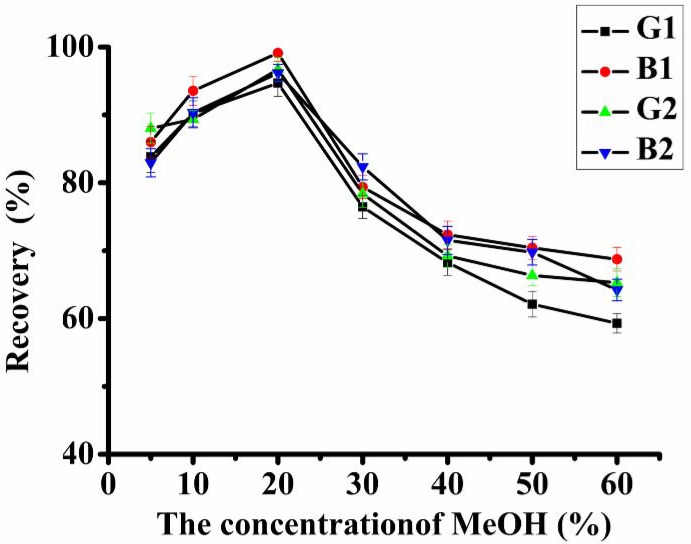
Effect of different loading solvents on the efficiency of IAC (aflatoxins spiked in peanut, 5 μg·kg^−1^ each).

The loading procedure largely depends on the physicochemical properties of the agricultural products that are contaminated with aflatoxins. The products containing high levels of lipids and nutritional compounds usually require a specific pre-treatment followed by more extensive purification methods than those with lower content of these components [13,14]. As aflatoxins dissolve in moderately polar solvents, different concentrations of MeOH (5%, 10%, 20%, 30%, 40%, 50%, 60%, v/v) were prepared to purify aflatoxins on the immunoaffinity column from the agri-products. The highest recovery was observed when 20% MeOH was used as loading solvent. In Figure 2, when the concentration of MeOH was less than 20%, the recovery of aflatoxins increased with the increasing concentrations of MeOH. The recovery of analytes was affected by the solubility of aflatoxins in organic solvents. Then, the recovery of aflatoxins decreased above 80% by increasing MeOH from 20%. IAC cleanup is based on the immunological reaction, which is the reversible association between MAb 1C11 and the corresponding antigens with the binding forces involve hydrogen bonds and hydrophobic binding. Organic solvents showed potent effects on the retention of AFT by MAb 1C11 in the IAC column. Our previous results showed that hydrogen bonding and hydrophobic interactions formed by Ser-H49 and Phe-H103 between the antibody with the haptens played the most important roles in the retention of the IAC column [15]. The results indicated that 20% MeOH solutions should be selected as the loading solvent without reducing analyte recovery.

### 2.3. Washing Condition

**Figure 3:**
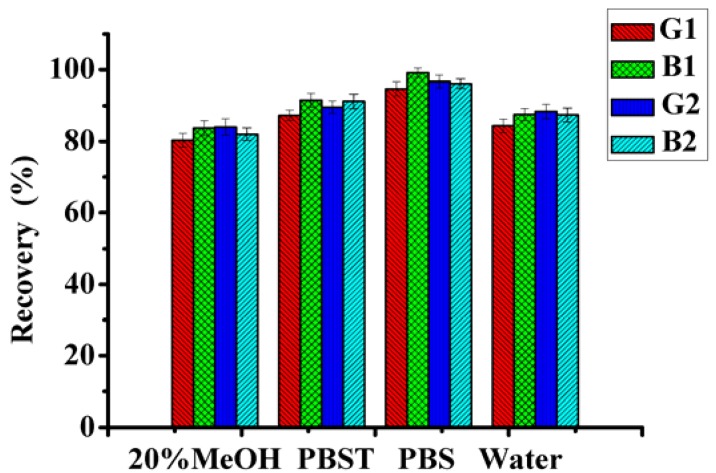
Effect of different washing solvents on the efficiency of IAC (aflatoxins spiked in peanut, 5 μg·kg^−1^ each).

Due to the high fat and protein levels in oilseeds (peanut, soybean, sunflower, *etc.*), sample matrices always cause high background in analysis chromatograms. In order to increase sensitivity and obtain a better chromatogram, various organic solvents were chosen. The following washing media were tested: water, MeOH/water (20:80, v/v), PBS, and PBST (Tween 20/PBS, 0.5:99.5, v/v). In Figure 3, the experimental results indicated that Tween 20 and PBS had significant effects on the washing efficiency. 

The recovery of the analytes fro the peanut samples using PBS solvent without Tween 20 were 94.7 ± 2.0%, 99.1 ± 1.2%, 96.7 ± 1.7% and 96.2 ± 1.3% for AFG_1_, AFB_1_, AFG_2_, and AFB_2_, respectively. Tween 20, which is a nonionic detergent, is commonly used in immunoassays to reduce nonspecific binding and improve sensitivity [16]. With the addition of Tweenm 20, the lower recovery of PBST might be caused by nonspecific hydrophobic interactions between detergent and the lipophilic aflatoxin molecules in the aqueous system. The results showed that PBS was the washing solvent with the best recovery of AFT.

### 2.4. Elution Conditions

Selection of the appropriate elution conditions was made attending to the recovery of the analytes, the volume needed for an acceptable recovery, and the compatibility with the analytical method [17,18]. Despite the fact that the use of acidic, basic buffers or solutions with high ionic strength has been reported, organic solvent/water mixtures such as MeOH/water, acetonitrile/water, ethanol/water have often achieved the best recovery for small molecules. As shown in Table 1, the best recovery (>80%) with less matrix interferences was obtained with methanol and ethanol.

**Table 1:** Effect of different organic solvents on the recovery of AFT.

Solvent ^a^	Solvent polarity scale	Recovery ^b^ (%)			
		AFB_1_	AFB_2_	AFG_1_	AFG_2_
Acetonitrile	0.895	76.5 ± 1.6	70.4 ± 3.7	69.1 ± 4.5	70.5 ± 2.7
Methanol	0.857	99.1 ± 1.3	96.2 ± 1.3	94.7 ± 2.0	96.7 ± 1.7
Ethanol	0.853	89.0 ± 6.8	83.5 ± 5.2	81.5 ± 7.6	82.3 ± 7.5

^a^ 5.0 g peanut sample containing 25 ng of AFG_1_, AFB_1_, AFG_2_ and AFB_2_ respectively, was subjected to the procedure described in Section 3.4; ^b^ The report data are the mean ± SD.

**Figure 4:**
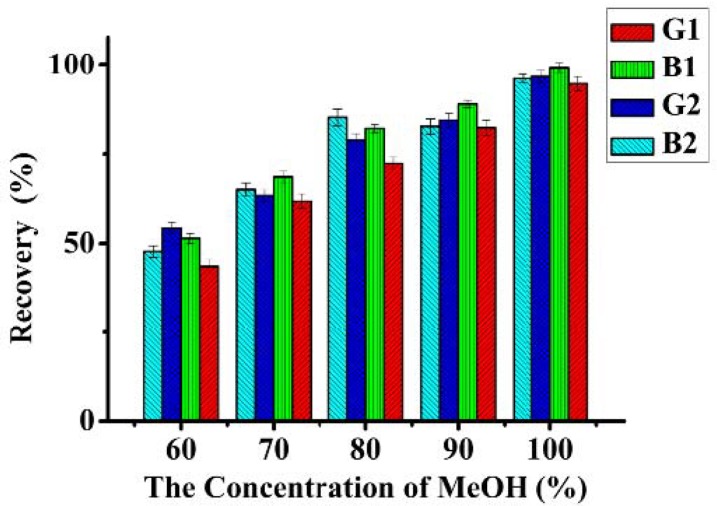
Effect of different eluting solvents on the efficiency of IAC (aflatoxins spiked in peanut, 5 μg·kg^−1^ each).

The high recoveries of methanol and ethanol showed that the appropriate solvent polarity might determine the elution efficiency [19]. In this experiment, different concentrations of MeOH were prepared for washing aflatoxins from IAC. After loading 10 mL of standard aflatoxins solutions (1.0 ng·mL^−1^), the IAC was washed with 5 mL PBS, then eluted with 1.0 mL MeOH/water (50%, 60%, 70%, 80%, 90%, 100%, v/v) collecting the eluate fractions. With the increase of MeOH concentration, the recovery of AFT in peanut samples increased dramatically. Figure 4 shows that the highest recovery was observed when 100% MeOH was used as eluting solvent. Elution solutions with high content of MeOH, which may cause damage to MAb 1C11, can decrease the number of times the IAC can be reused. However, due to the evaporation and the pre-column derivatization steps in the method, water contained in the eluate solutions made the clean-up procedure more time-consuming and gave less side products. IAC column recycle procedures including tedious steps were, however, not anticipated for daily use even if the columns could be reused many times. Thus, MeOH was selected as the elution solvent.

### 2.5. Binding Capacity

**Figure 5:**
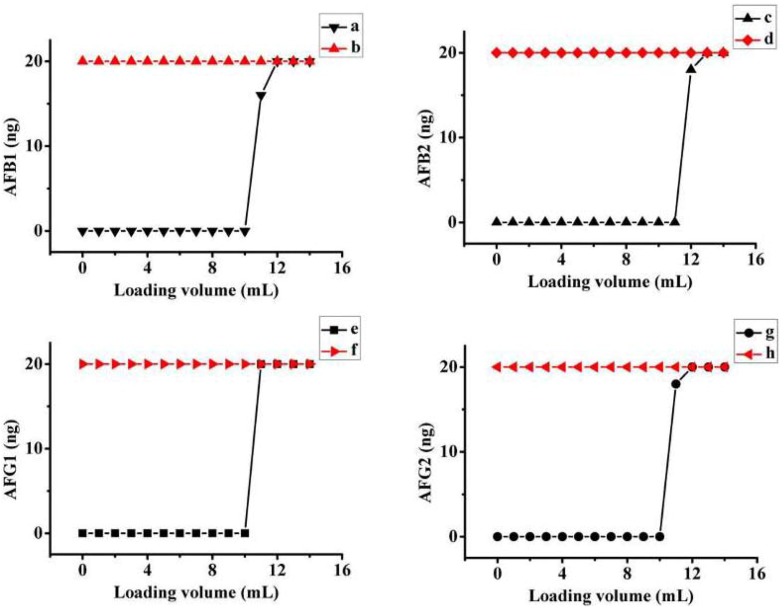
Elution profiles for the continuous loading AFB_1_, AFB_2_, AFG_1_, AFG_2_ standard solution at concentration of 20 ng·mL^−1^ onto IAC (**a**, **c**, **e**, **g**) and the blank column with amino-silica gel microparticle (no conjugation with antibody MAb 1C11) (**b**, **d**, **f**, **h**).

Under the optimal IAC conditions for aflatoxins described above, loading, washing and elution solutions were MeOH/water (20%, v/v), PBS and MeOH, respectively. The flow rates of the loading, washing and elution steps were 1 mL·min^−1^, 2.0 mL·min^−1^ and 1.0 mL·min^−1^, respectively [20]. To evaluate the maximum capacity of the IAC, the binding capacity was determined by overloading with 20 mL of AFB_1_, AFB_2_, AFG_1_, AFG_2_, standard solution (20 ng·mL^−1^). The results (Figure 5) indicated that the columns containing 0.25 mL immunosorbent showed a binding capacity of 200, 220, 200 and 200 ng for AFB_1_, AFB_2_, AFG_1_, AFG_2_, respectively.

### 2.6. Linearity, Detection and Quantification Limits

Under the above optimized conditions, the method was validated for linearity, precision, detection limit, recovery, inter-assay and intra-assay deviation. The calibration curve was constructed using the chromatographic peaks of aflatoxins at different increasing levels. The linearity of the above-mentioned ranges was established by analysis of different calibration levels in three replicates. The results indicated that the standard calibration graphs were linear for AFB_1_ and AFG_1_ (0.50–100.0 ng·mL^−1^), AFB_2_ and AFG_2_ (0.25–50.0 ng·mL^−1^), the correlation coefficients (*r*^2^) 0.999 for all aflatoxins. The limit of detection (LOD) and the quantification (LOQ) were determined by the signal-to-noise approach, defined as those levels resulting in signal-to-noise ratios of 3 and 10, respectively. The analytic response and the chromatographic noise were both measured from the chromatogram of a blank sample extract to which between 1.5 and 15 μL of aflatoxins solution (0.50 μg·mL^−1^ for AFG_1_, AFB_1_, AFG_2_, AFB_2_, respectively) had been added. LOD and LOQ of the method were calculated to be 0.03 and 0.10 μg·kg^−1^, 0.07 and 0.25 μg·kg^−1^, 0.05 and 0.18 μg·kg^−1^, 0.09 and 0.30 μg·kg^−1^, for AFG_1_, AFB_1_, AFG_2_, AFB_2_, respectively. LODs and LOQs, which were obtained with IAC, were well below the current maximum levels for agri-product, as established by the European Commission [2 μg·kg^−1^ for AFB_1_ and 4 μg·kg^−1^ for total aflatoxins (B_1_ + B_2_ +G_1_ + G_2_)] [4]. The recoveries, and intra-, inter-day, inter-laboratory relative standard deviations (RSDs) were calculated with the aflatoxins spiked at six different concentration levels in tea, oil and peanut, respectively. In Table 2, Table 3, acceptable precision as reflected from the relative standard deviations (RSDs) values for intra-day study (1.7%–4.4%), inter-day (1.4%–4.0%) and inter-laboratory (1.9%–3.5%) were found. 

**Table 2:** Results for aflatoxins in validation study for peanut samples.

Spiked	Intra-day repeatability				Inter-laboratory reproducibility ^a ^				Mean recovery (%) ± RSD (%)			
amount	(RSD%, n = 6)				(RSD%, n = 6)				(n = 12)			
(μg·kg^−1^)	AFB_1_	AFG_1_	AFB_2_	AFG_2_	AFB_1_	AFG_1_	AFB_2_	AFG_2_	AFB_1_	AFG_1_	AFB_2_	AFG_2_
0.30	-	-	2.9	3.1	-	-	3.2	3.5	-	-	98.3 ± 2.7	95.6 ± 3.2
0.50	3.2	2.7	2.4	1.9	2.5	3.1	1.8	1.9	96.1 ± 4.2	90.1 ± 2.7	94.2 ± 3.1	93.3 ± 2.1
1.00	2.6	1.8	1.8	2.2	2.4	2.3	1.5	2.1	104.4 ± 2.7	93.1 ± 1.9	98.2 ± 1.9	97.5 ± 2.5
2.00	2.1	1.7	-	-	1.9	2.0	-	-	101.6 ± 2.5	98.1 ± 2.2	-	-
4.00	2.7	2.1	1.8	2.4	2.6	2.9	2.3	2.8	99.9 ± 2.2	98.0 ± 2.1	98.0 ± 1.6	96.7 ± 2.1
10.00	2.5	2.2	2.0	2.1	2.5	2.2	2.0	2.1	98.2 ± 2.0	98.2 ± 1.9	98.7 ± 1.6	97.1 ± 1.6
25.00	2.0	2.3	2.4	2.5	2.8	2.6	2.5	2.7	98.9 ± 1.8	98.5 ± 1.9	97.8 ± 2.0	97.0 ± 2.0

^a^ All laboratories which participated in the exercise for determination of method reproducibility included Key Laboratory of Detection for Mycotoxins, Ministry of Agriculture, Wuhan, China; Shandong Analysis and Test Center, Shandong Academy of Agricultural Sciences, Jinan, China; Beeproduct Quality Supervision and Test Center, Ministry of Agriculture, Beijing, China; Center of Quality Standards & Testing Technology for Agriculture, Henan Academy of Agricultural Sciences, Zhengzhou, China; College of Food Science, Southwest University, Chongqing, China; Hangzhou Center for Inspection and Testing for Quality and Safety of Agricultural and Genetically Modified Products, Ministry of Agriculture, Hangzhou, China.

**Table 3:** Results for aflatoxins in the precision and recoveries study for oil and tea samples.

Sample	Spiked	Intra-day repeatability				Inter-day reproducibility				Mean recovery (%) ± RSD (%)			
	amount	(RSD%, n = 6)				(RSD%, n = 6)				(n = 12)			
	(μg·kg^−1^)	AFB_1_	AFG_1_	AFB_2_	AFG_2_	AFB_1_	AFG_1_	AFB_2_	AFG_2_	AFB_1_	AFG_1_	AFB_2_	AFG_2_
Oil	0.30	-	-	4.4	4.2	-	-	3.3	4.0	-	-	95.0 ± 2.8	94.1 ± 3.1
	0.50	2.9	3.2	3.2	3.0	2.5	2.5	2.6	1.4	98.7 ± 2.0	99.5 ± 2.3	96.8 ± 2.2	97.0 ± 1.6
	1.00	3.7	3.2	3.1	4.1	3.0	2.4	2.5	2.5	98.8 ± 2.3	99.2 ± 2.3	97.0 ± 2.2	98.3 ± 2.6
	2.00	2.6	2.7	-	-	2.0	2.8	-	-	99.0 ± 1.7	98.8 ± 2.1	-	-
	4.00	2.9	3.5	3.1	2.2	2.4	2.5	2.6	2.6	95.4 ± 2.5	98.0 ± 2.2	98.4 ± 2.0	97.9 ± 1.7
	10.00	3.0	3.4	3.3	3.5	2.6	2.3	2.4	3.0	95.2 ± 2.8	97.3 ± 1.9	99.3 ± 2.2	98.0 ± 2.6
	25.00	2.3	2.7	3.4	2.2	3.4	3.5	2.7	2.6	98.3 ± 2.2	96.4 ± 1.7	97.5 ± 2.2	96.1 ± 2.3
Tea	0.30	-	-	2.8	3.6	-	-	2.2	2.7	-	-	97.6 ± 1.8	98.4 ± 2.3
	0.50	3.3	3.2	3.3	3.4	2.5	3.7	2.3	3.0	98.6 ± 2.2	99.0 ± 2.6	98.3 ± 2.0	97.4 ± 2.6
	1.00	2.3	2.5	3.8	3.7	3.1	3.1	3.1	2.8	98.0 ± 2.0	99.4 ± 2.0	98.2 ± 2.6	98.9 ± 2.6
	2.00	2.8	2.6	-	-	2.4	2.2	-	-	98.9 ± 2.0	97.6 ± 1.9	-	-
	4.00	2.8	2.6	2.8	3.6	2.4	2.2	2.2	2.7	98.9 ± 2.0	97.6 ± 1.9	97.6 ± 1.8	98.4 ± 2.3
	10.00	3.3	2.9	3.0	2.8	3.2	2.6	2.7	2.0	98.2 ± 2.3	98.4 ± 2.1	98.4 ± 2.3	97.8 ± 2.2
	25.00	2.5	2.9	2.7	2.8	2.5	2.9	2.7	2.8	99.0 ± 1.8	98.8 ± 1.8	98.2 ± 1.8	98.5 ± 2.1

**Figure 6:**
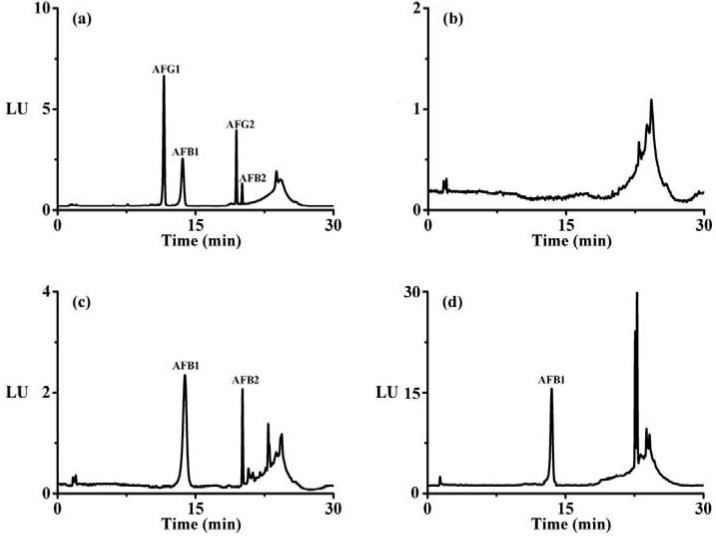
Chromatograms of aflatoxins in the peanut sample (**a**, aflatoxins spiked at 1 μg·kg^−1^ each), the blank peanut sample (**b**), naturally contaminated samples of vegetable oil (**c**) and tea (**d**).

Results also showed that the recovery values were in the range of 90.1%–104.4%. Additionally, blank samples were extracted and analyzed to assess the potential interferences. In Figure 6, no interfering peaks from the sample matrix were observed at the retention time of AFG_1_, AFB_1_, AFG_2_, AFB_2_, which indicated the good practicability of the amino-silica gel microparticle-based immunoaffinity column clean-up method.

### 2.7. Analysis of Real Agri-Product Samples

For further validation of the applicability, the IAC method was applied for the analysis of agri-products, including peanut, vegetable oil and tea. Analysis of aflatoxins of these samples, especially peanut and tea, are not simple due to the presence of high oil content, proteins, pigments or polyphenols which could be co-extracted with aflatoxins. In Figure 6, typical chromatograms of aflatoxins in naturally contaminated oil and tea sample were presented. Results of the determination of AFT in these samples were summarized in Table 4. Among the agri-products analysed, peanut samples were contaminated with high amount of aflatoxins ranging from 0.49–20.79 μg·kg^−1^. These samples were contaminated mainly with AFB_1_, AFB_2_ and to a lesser extent by AFG_1_, AFG_2_. The levels of AFB_1_, AFB_2_ and AFG_2_ range from 0.27–12.63, 0.06–3.88 and 0.02–0.22 μg·kg^−1^, respectively. Despite of the high incidence of contamination by AFT, the vegetable oils had relatively low levels of AFT, ranging from 0.27–0.89 μg·kg^−1^. Higher levels of AFT were found in peanuts and its related vegetable oils which were pressed from oilseeds. The results indicated that the contamination of AFT in vegetable oils was linked to the quality, storage conditions and processing treatment of raw materials. The traditional method of drying these peanut items on the ground in the open air in poor hygienic conditions promoted the growth of moulds and production of mycotoxins. It was suggested that dry and clean practices must be in place in the preparation of these commodities.

**Table 4:** Results for the determination of AFT in agri-products.

Sample	No. of	No. positive	Mean ^a^	Range	Incidence	Average aflatoxin (μg·kg^−1^)			
	Analysed samples	analysed samples	(μg·kg^−1^)	(μg·kg^−1^)	(%)	AFB_1_	AFB_2_	AFG_1_	AFG_2_
Peanut	52	14	5.20 ± 0.05	0.49–20.79	26.9	4.15	1.01	ND ^b^	ND
Vegetable oil	25	7	0.52 ±0.01	0.27–0.89	28.0	0.46	0.06	ND	ND
Tea	19	1	7.80 ± 0.03	-	5.3	7.80	ND	ND	ND

^a^ Results are represented as means (n = 6) ± SD; ^b^ ND, not detected.

## 3. Experimental

### 3.1. Chemicals and Materials

The standards of AFB_1_, AFG_1_, AFB_2_ and AFG_2_ were purchased from Supelco (Bellefonte, PA, USA). Aflatoxin stock solutions were made up in acetonitrile at concentrations of 2.0 μg·mL^−1^ for AFB_1_ and AFG_1_, and 1.0 μg·mL^−1^ for AFB_2_ and AFG_2_. Standard solutions were prepared before use by diluting stock solution with mobile phase. These solutions were stored at 4 °C in the darkness.

Amino-silica gel microparticles (250 ± 20 μm) were obtained from Wuhan University Chemical Factory (Wuhan, China). 1-Ethyl-(3-dimethylaminopropyl)-carbodiimide hydrochloride (EDC·HCl) was purchased from Acros Organics (Gell, Belgium). Phosphate-buffered saline (PBS) tablets and Tween 20 were purchased from Sigma-Aldrich Co. (St. Louis, MO, USA). MAb 1C11 against aflatoxins was prepared in our laboratory [21]. The empty column and frit were purchased from Shenzhen Biocomma Biotech Co. Ltd. (Shenzhen, China). All other chemicals were analytical grade and supplied by Sinopharm Chemical Reagent Co. Ltd. (Shanghai, China). The water used for chromatography was purified on a Milli-Q System (Waters, Milford, MA, USA) and then filtered through 0.22 μm membrane filters.

### 3.2. Instrumentation

A FT-Near infrared (NIR) spectrometer (Bruker Optics Inc., Ettlingen, Germany), fluorescence spectrophotometer (F4500, Hitachi Tech., Tokyo, Japan), and ultraviolet spectrum (Molecular Devices Corp., Sunnyvale, CA, USA) were used in this study. Quantitative analyses were performed on an Agilent 1100 HPLC system (Agilent Tech., Santa Clara, CA, USA). The ultrasonic-assisted extraction was carried out by a KQ-800KDE ultrasonic device (Kunshan Ultrasound Instrument Company, Kunshan, China).

### 3.3. Preparation of IAC Columns

MAb 1C11 were purified from ascites by a classic method of saturated ammonium sulphate solution as described previously [22]. The immunoaffinity column was prepared by coupling MAb 1C11 with the amino-silica gel microparticles according to the Wuhan University Chemical Factory instructions and related literature [23]. Amino-silica gel microparticles (1.0 g) were swollen thoroughly in 0.05 mol·L^−1^ hydrochloric acid (5.0 mL), and then washed with methanol (8.0 mL) and pure water (12.0 mL). The obtained amino-silica gel was dissolved in PBS (5 mL) and then was mixed with 0.5 mg·mL^−1^ MAb 1C11 solution (2 mL) at 4 °C. After adding EDC·HCl (60 mg), the mixture pH was adjusted to 6.0 by adding 0.006 mol·L^−^^1^ KH_2_PO_4_, and then the solution was gently agitated for 24 h at 4 °C. The reaction yielded 2.6 mL of precipitated conjugates and 8.0 mL supernatant. A volume of 0.26 mL conjugated amino-silica gel microparticles was accurately measured and packed in the immunoaffinity column (1 mL). Then the conjugates were naturally precipitated and the free immunoglobulin (IgG) in the supernatant solution was measured by an UV-Vis spectrophotometer and calculated based on the UV absorption difference between 280 nm and 260 nm. The formula used for the calculation was [24]:

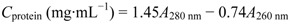


### 3.4. Sample Extraction

For the method development and the validation, a total of 96 samples of agri-products (peanut, vegetable oil and tea, at least 3.0 kg) were collected from local markets and supermarkets. The samples were stored under ventilated and dry conditions until analysis. Homogenization of the samples was accomplished by thorough stirring (oil samples) and pulverizing (other samples) using a mechanical mortar. Peanut kernels and tea samples finely ground with a blender until they could pass through a no. 20 sieve, then the samples were rendered into a paste and stored at 4 °C in suitable glass container before analysis.

#### 3.4.1. Extraction of Aflatoxins from Peanut

The samples were prepared according to the procedure of Stroka [25] with slight modifications. Sample (25.0 g) was placed in a 250 mL Erlenmeyer flask and extracted in an ultrasonic bath with methanol/water (80% v/v, 75 mL) containing 4% NaCl at 50 °C for 10 min. Then the extract was filtered through double-layered filter paper. The filtrate (20 mL) was mixed with petroleum ether (10 mL) and vortexed for 1 min. After standing, the lower methanol-water solution (15 mL) was transferred and diluted with pure water (40 mL), blended and filtered through an organic membrane (0.45 μm).

#### 3.4.2. Extraction of Aflatoxins from Vegetable Oil

Sample (25.0 g) was placed in a 250 mL Erlenmeyer flask, then mixed with methanol/water (80% v/v, 75 mL) containing 4% NaCl and petroleum ether (50 mL) and vortexed for 2 min. After standing, the lower methanol-water solution (15 mL) was transferred and diluted with pure water (40 mL), blended and filtered through an organic membrane (0.45 μm).

#### 3.4.3. Extraction of Aflatoxins from Tea

Sample (25.0 g) was placed in a 250 mL Erlenmeyer flask and extracted with methanol/water (80% v/v, 75 mL) containing 4% NaCl under ultrasound at 50 °C for 10 min. Then the extract was filtered through double-layered filter paper. The filtrate (15 mL) was diluted with pure water (40 mL), blended and filtered through an organic membrane (0.45 μm).

### 3.5. IAC Purification of Sample Extract

Under the optimized conditions, immunosorbent (0.25 mL) was measured and packed in a 1 mL column. The column was preconditioned by PBS (10 mL) prior to sample application and kept under buffer during the experiment. The extracts obtained (8 mL) were passed through immunoaffinity columns, then washed with PBS (5 mL) and eluted with methanol (2 mL) into glass tubes. The flow rates of the loading, washing and eluting steps were 1 mL·min^−1^, 2.0 mL·min^−1^ and 1.0 mL·min^−1^, respectively. The eluate was dried with nitrogen at 60 °C. The purified extract was then derivatized with trifluoroacetic acid (100 μL) in hexane (200 μL) at 40 °C for 20 min [14,20]. The reactant was dried under nitrogen flow at 50 °C. The residues were dissolved in acetonitrile/water (15% v/v, 1 mL) and injected to the HPLC system for analysis.

### 3.6. HPLC-FLD Analysis

HPLC-FLD analysis was performed on an Agilent 1100 system equipped with a fluorescence detector and a separation column (Shiseido, Tokyo, Japan. C_18_, 150 mm × 4.6 mm I.D., 5 µm particle size) was used for separation. All the HPLC analysis was performed at a flow rate of 1.0 mL min^−^^1^. The mobile phases were acetonitrile (A) and water (B). After an isocratic step at 15% of A for 6 min, then was increased up to 17% A for 2 min, then held to 25% A from 8.1–14.0 min. A was decreased to 15% for 8 min, then held to 15% A from 22.1–26.0 min. The injection volume was 20 μL. The detection of the excitation and emission wavelengths were fixed at 360 nm and 440 nm, respectively.

## 4. Conclusions

The developed aflatoxin immunoaffinity column using the specific MAb 1C11 against the analytes to couple with amino-silica gel microparticles, showed affinity and specificity towards aflatoxins. Effects of experimental parameters on the IAC procedure of the analytes have been evaluated, and the optimal conditions obtained in this work showed good extraction efficiency and recovery. Applied to the analysis of real agri-products, including peanut, vegetable oils and tea, it showed that cleaning-up of the extracts using IAC is effective in removing unwanted interfering components. The developed IAC cleanup procedure coupled with HPLC analysis could be hopefully used as an alternative method for the determination of aflatoxins in complex actual samples.

## References

[1] Fu A.B., Huang X.X., Min S.G. (2008). Rapid determination of aflatoxins in corn and peanuts. J. Chromatogr. A.

[2] Cervino C., Knopp D., Weller M.G., Niessner R. (2007). Novel aflatoxin derivatives and protein conjugates. Molecules.

[3] Armstrong B., Miller A.B. (1993). Some Naturally Occurring Substances: Food Items and Constituents, Heterocyclic Aromatic Amines and Mycotoxins. IARC Monographs on the Evaluation of the Carcinogenic Risks to Humans.

[4] Barroso J.M. (2010). Commission Regulation (EU) No 165/2010 Amending Regulation (EC) No 1881/2006Setting Maximum Levels for Certain Contaminants in Foodstuffs as Regards Aflatoxins Text with EEA Relevance.

[5] Beaver R.W., Wilson D.M., Trucksess M.W. (1990). Comparison of postcolumn derivatization-liquid chromatography with thin-layer chromatography for determination of aflatoxins in naturally contaminated corn. J. AOAC Int..

[6] Gomez-Catalan J., Pique E., Falco G., Borrego N., Rodamilans M., Llobet J.M. (2005). Determination of aflatoxins in medicinal herbs by HPLC. An efficient method for routine analysis. Phytochem. Anal..

[7] Edinboro L.E., Karnes H.T. (2005). Determination of aflatoxin B1 in sidestream cigarette smoke by immunoaffinity column extraction coupled with liquid chromatography/mass spectrometry. J. Chromatogr. A.

[8] Cervino C., Asam S., Knopp D., Rychlik M., Niessner R. (2008). Use of isotope-labeled aflatoxins for LC-MS/MS stable isotope dilution analysis of foods. J. Agric. Food Chem..

[9] Lee N.A., Wang S., Allan R.D., Kennedy I.R. (2004). A rapid aflatoxin B1 ELISA: development and validation with reduced matrix effects for peanuts, corn, pistachio, and Soybeans. J. Agric. Food Chem..

[10] Sheibani A., Tabrizchi M., Ghaziaskar H.S. (2008). Determination of aflatoxins B1 and B2 using ion mobility spectrometry. Talanta.

[11] Shelver W.L., Larsen G.L., Huwe J.K. (1998). Use of an immunoaffinity column for tetrachlorodibenzo-p-dioxin serum sample cleanup. J. Chromatogr. B.

[12] Kussak A., Andersson B., Andersson K. (1995). Immunoaffinity column clean-up for the high-performance liquid chromatographic determination of aflatoxins B1, B2, G1, G2, M1 and Q1 in urine. J. Chromatogr. B.

[13] Senyuva H.Z., Gilbert J. (2010). Immunoaffinity column clean-up techniques in food analysis: A review. J. Chromatogr. A.

[14] Khayoon W.S., Saad B., Yan C.B., Hashim N.H., Ali A.S.M., Salleh M.I., Salleh B.  (2010). Determination of aflatoxins in animal feeds by HPLC with multifunctional column clean-up. Food Chem..

[15] Li X., Li P.W., Zhang Q., Li Y.Y., Zhang W., Ding X.X. (2012). Molecular characterization of monoclonal antibodies against aflatoxins: A possible explanation for the highest sensitivity. Anal. Chem..

[16] Lee J.K., Ahn K.C., Park O.S., Kang S.Y., Hammock B.D. (2001). Development of an ELISA for the detection of the residues of the insecticide imidacloprid in agricultural and environmental samples. J. Agric. Food. Chem..

[17] Sanvicens N., Moore E.J., Guilbault G.G., Marco M.P. (2006). Determination of haloanisols in white wine by immunosorbent solid-phase extraction followed by enzyme-linked immunosorbent assay. J. Agric. Food Chem..

[18] Rejeb S.B., Cléroux C., Lawrence J.F., Geay P.Y., Wu S.G., Stavinski S. (2001). Development and characterization of immunoaffinity columns for the selective extraction of a new developmental pesticide: Thifluzamide, from peanuts. Anal. Chim. Acta.

[19] Catalán J., López V., Pérez P., Martín-Villamil R., Rodríguez J.G. (1995). Progress towards a generalized solvent polarity scale: The solvatochromism of 2-(dimethylamino)-7-nitrofluorene and its homomorph 2-fluoro-7-nitrofluorene. Liebiges Ann..

[20] Liu X.M., Wang J., Li F.Q., Ji R., Chen J.D., Yao X.P, Luo H.D, Zhang H.Y. (2006). Determination of Aflatoxins B_1_, B_2_, G_1_, G_2_ in Foods.

[21] Zhang D.H., Li P.W., Zhang Q., Zhang W., Huang Y.L., Ding X.X., Jiang J. (2009). Production of ultrasensitive generic monoclonal antibodies against major aflatoxins using a modified two-step screening procedure. Anal. Chim. Acta.

[22] Russo C., Callegaro L., Lanza E., Ferrone S. (1983). Purification of IgG monoclonal antibody by caprylic acid precipitation. J. Immunol. Methods.

[23] Ke Y.J., Li P.W., Zhang W., Liu H.H., Li S.Y. (2007). The conjunction of several macromolecule microsphere with rabbit immune globin and the optimization of the conjunction conditions. J. Hubei Univ..

[24] Zhao M.P., Liu Y., Li Y.Z., Zhang X.X., Chang W.B. (2003). Development and characterization of an immunoaffinity column for the selective extraction of bisphenol A from serum samples. J. Chromatogr. B.

[25] Stroka J., Anklam E., Jörissen U., Gilbert J. (2000). Immunoaffinity column cleanup with liquid chromatography using post-column bromination for determination of aflatoxins in peanut butter, pistachio paste, fig paste, and paprika powder: collaborative study. J. AOAC Int..

